# Retraction: Seedbank persistence of four summer grass weed species in the northeast cropping region of Australia

**DOI:** 10.1371/journal.pone.0327065

**Published:** 2025-06-25

**Authors:** The PLOS One Editors

The *PLOS One* Editors retract this article [1] because it was identified as one of a series of submissions for which we have concerns about competing interests and peer review. We regret that the issues were not addressed prior to the article’s publication.

The authors did not agree with retraction.
